# Colour vision in ants (Formicidae, Hymenoptera)

**DOI:** 10.1098/rstb.2021.0291

**Published:** 2022-10-24

**Authors:** Ayse Yilmaz, Johannes Spaethe

**Affiliations:** ^1^ Department of Biology, Lund Vision Group, Lund University, 223 62 Lund, Sweden; ^2^ Behavioral Physiology and Sociobiology (Zoology II), Biocenter, University of Würzburg, Sanderring 2, 97070 Würzburg, Germany

**Keywords:** insects, colour vision, dichromacy, trichromacy, opsins, spectral sensitivity

## Abstract

Ants are ecologically one of the most important groups of insects and exhibit impressive capabilities for visual learning and orientation. Studies on numerous ant species demonstrate that ants can learn to discriminate between different colours irrespective of light intensity and modify their behaviour accordingly. However, the findings across species are variable and inconsistent, suggesting that our understanding of colour vision in ants and what roles ecological and phylogenetic factors play is at an early stage. This review provides a brief synopsis of the critical findings of the past century of research by compiling studies that address molecular, physiological and behavioural aspects of ant colour vision. With this, we aim to improve our understanding of colour vision and to gain deeper insights into the mysterious and colourful world of ants.

This article is part of the theme issue ‘Understanding colour vision: molecular, physiological, neuronal and behavioural studies in arthropods’.

## 1. Introduction

Colour vision is the ability of an animal to distinguish between objects based on their spectral composition, regardless of the relative light intensity [1,2]. Due to their diverse ecology, often stereotyped yet manifold behaviours and well-characterized genetic and anatomic visual structures, insects became an attractive system to understand the mechanisms underlying (colour) vision. Like many other animals, insects use colour information (besides other sensory modalities), during both day and night, to guide their behaviour, locate specific habitats, identify conspecifics and orient by means of celestial and terrestrial landmarks [3]. This capacity allows them to avoid problems imposed by varying illumination which can change drastically in the course of a day or between different habitats [4,5].

Colour vision involves different physiological and neuronal processing stages that mainly require the comparison of the outputs of at least two spectrally distinct photoreceptor (PR) types within the retina [1]. The absolute limit for colour vision is set by light intensity [6], but in insects several other factors can modify PR sensitivity, such as screening pigments, filtering pigments and rhabdom structure (e.g. open versus fused, or stacked versus elongated PR cells) [3,7]. In the early processing stage, signals may be compared already at the PR level [8,9] and by means of colour-opponent neurons in the optic lobes [10–12]. Higher order neuronal processing is further required to build up a percept of colour and finally to induce a behavioural response [11,13,14].

In this review, we aim to provide a brief overview of the colour vision capabilities of one of the most ecologically important insect groups, the ants [15]. For this, we compiled studies that address physiological, molecular, neuronal and behavioural aspects of ant colour vision, and, when reasonable, compared them with other insect species. In the strict sense, colour vision comprises two aspects, (i) the perception of chromatic information irrespective of brightness and (ii) spatial vision, where colour is associated with an object or is restricted in its spatial dimension [16]. Since not all studies follow this definition of (true) colour vision, we also included those which investigated colour vision in a broader sense, e.g. wavelength-specific behaviour [17].

## 2. Diversity of ant visual environments

Ants occupy a wide range of habitats, including grasslands, deserts and tropical rainforests, on all continents except Antarctica. Their ability to colonize such diverse habitats is attributed to their outstanding social organization as superorganisms, and to their capability to efficiently exploit a wide range of food sources as herbivores, predators and scavengers [15]. Their visual environments vary in spectral characteristics among habitats or within the same habitat over time depending on its structure, the time of day or season [18], which may have led to numerous adaptions of the visual system [19–21] and the use of particular spectral information [4]. Many ant species are active during the day (diurnal), when light level reliably supports vision. However, a considerable number of species are active at twilight or under dim light conditions [15,20,22–24]. Nocturnal (night active) ants often prioritize olfactory cues over visual cues due to a low visual signal-to-noise ratio at night, but would still benefit from visual information to locate food, recognize conspecifics and navigate back to their nests [24,25]. To accomplish this, some species increased their light sensitivity by modifying the optical and neuronal properties of the visual system [19,20,23]. Among insects, some moths [26] and bees [27] are known to use colour vision under dim light conditions. Although not yet demonstrated, such capabilities may be present in ant species, which live in a visually rich environment and are active under dim light conditions.

## 3. Eye morphology and spectral sensitivity of photoreceptors

Like most hymenopterans, ants have apposition compound eyes with several dozens to hundreds of ommatidia per eye. The number and morphological characteristics (i.e. lens diameter and rhabdom length) of ommatidia vary among species or even within castes or sexes of the same species depending on body size, activity rhythm (diurnal versus nocturnal) and ecological requirements (see above). For example, workers of the diurnal desert ants *Catagylphis bicolor* and *Camponotus detritus* have approximately 1300 ommatidia per eye [19], while this number in the fire ant *Solenopsis invicta* ranges between 48 in minor workers to 92 in major workers [28]. Such differences may also reflect the behavioural differences (e.g. diurnal versus nocturnal activity) of different species.

### (a) Early histological studies

**Figure 1.**
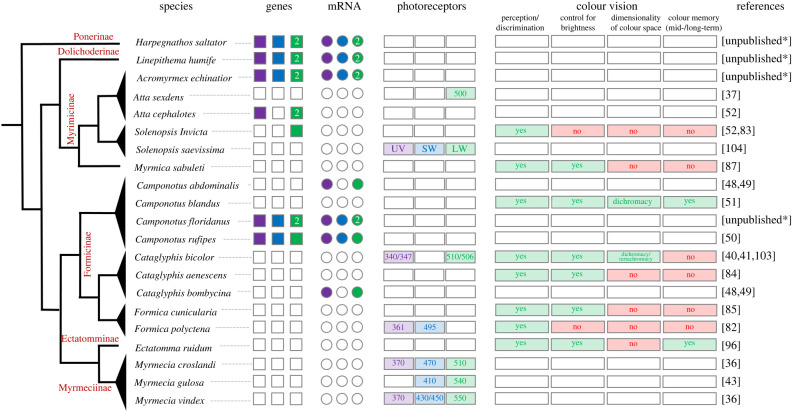
Compiled literature data of opsin genes, opsin mRNA expression, PR types and colour vision experiments from 21 ant species and six subfamilies. Numbers in boxes and circles indicate evidence for paralogous LW genes. When available, spectral sensitivity maxima are shown for each PR in the corresponding box. Colour vision experiments are categorized for different aspects, e.g. if true colour vision was tested (control for brightness), if the number of involved PRs was determined (dimension of colour appearance) and if the ants exhibited mid- or long-term colour memory. Empty boxes and circles indicate that data are not available. Cladogram based on Ward *et al.* [32–34]. UV, ultraviolet (purple); SW, short-wavelength (blue); LW, long-wavelength (green). *, unpublished Sequence data can be provided upon request.

Common to all investigated ant species and most other hymenopterans, each ommatidium comprises eight PR cells with long rhabdoms spanning the entire retina and one basal PR cell with a short rhabdomere (R1–R9) forming a central rhabdom [29]. Early histological studies used the radial migration of pigments in the PR cells during selective chromatic adaptation to determine different spectral receptor types and their arrangement within the ommatidium [29,30]. These studies are based on the phenomenon that the location of the pigments within the PR cell depends on the intensity and the spectral component of the incident light [27,31]. Cells, which are (over-) exposed to spectral light for which they are sensitive, respond with a migration of pigment granules towards the rhabdom to reduce the photon flux and avoid excess light damage. The pigment granules encase the rhabdom (more precisely, the rhabdomere of the respective PR cell), which function as a light guide, and absorb part of the photons. Contrary to bees, it was assumed that the PR cells of an ant ommatidium are not electrically coupled and that the pigment granules in adjacent PR cells move independently upon light adaptation [30]. Thus, chromatic illumination was expected to allow for a differentiation between spectrally distinct PR cells within the retina. Chromatic adaptation experiments coupled with electron microscopy techniques in *Formica polyctena* [30] and *Myrmecia gulosa* [29] revealed that the pigments in R1 and R5 cells, which lie opposite each other, moved selectively after ultraviolet (UV) light exposure. By contrast, the pigments in the remaining six PR cells were most sensitive to long-wavelength (LW) light (note that the sensitivity of the basal R9 cell has not been addressed). The presence of at least two PR types, which respond mostly to UV and green light, respectively, was later supported by electrophysiological and behavioural studies (see below; figure 1).

### (b) Electrophysiological studies

The spectral sensitivity of a PR determines the probability of capturing light of particular wavelengths [12,35]. Different electrophysiological techniques have been used to measure the spectral sensitivities of PRs in ants [36]. The most common method is electroretinography (ERG), which measures the sum response of PR and lamina neurons in response to (monochromatic) light. ERG measurements are usually performed in intact immobilized insects using a thin glass (recording) electrode (or silver-silver chloride wire, [37]) that is inserted in the retina while applying a short light pulse, usually lasting from a few milliseconds to seconds. An additional reference electrode is inserted in a unilluminated part of the body (i.e. head or thorax). During ERG measurements, the electrical activity of cells, i.e. voltage change, corresponds to the stimulus intensity and the sum of responding PRs and lamina neurons [35]. ERG measurements are typically performed in dark-adapted animals, but selective adaptation to certain spectral lights is used to saturate a subpopulation of PRs to make rare PR types visible, since those PRs are usually masked by the more dominant ones [36]. In contrast to ERG measurements, intracellular recording has been used to measure the spectral response of individual PR cells. A sharp recording electrode connected to a high impedance microelectrode amplifier is inserted directly into a PR axon in the retina using a micromanipulator [38]. This allows a direct quantification of the sensitivity of a PR, excluding the responses of optic lobe neurons.

Earlier studies using ERG and intracellular recordings found evidence for the presence of two different PR types that are most sensitive to LW (green) and short wavelengths (SW) (UV) of light (ERG: *Formica polyctena*, [31,39]; *Lasius niger*, *Formica cunicularia*, [40]; *Cataglyphis bicolor,* [41]; intracellular recordings: *Cataglyphis bicolor,* [42] and *Myrmecia gulosa*, [43], figure 1). One study revealed only one PR type with a maximal response at 500 nm in *Atta sexdens rubropilosa* [37], but the authors did not test wavelengths below 400 nm and thus most likely missed a UV-sensitive PR.

By contrast, a more recent electrophysiological study found three spectrally distinct PR types (with peak sensitivities to short, middle and LW) in the compound eye of two Australian ant species, the diurnal *Myrmecia croslandi* and the nocturnal *Myrmecia vindex* [36], suggesting a more diverse pattern of PR expression among ant eyes, including species with three spectrally distinct PRs. The authors suggested that *Myrmecia* ants might have retained trichromacy from their wasp-like ancestors [36], while the more derived ant genera *Formica* and *Cataglyphis* may have lost the blue light receptor over evolutionary time. Although the presence of three visual opsin genes in most ants investigated so far (see below) supports this hypothesis, an earlier study [43] found only two (blue- and green-light-sensitive) PR types in another *Myrmecia* species (see also figure 1), clearly highlighting the need for more physiological data.

The absence of a blue light-sensitive PR is common to many ants and has been noted for several other insect taxa. For example, the loss of the blue-light-sensitive PR has been shown in the basal hymenopteran *Sirex noctilio* [44] and in most beetle species [21]. The functional significance of this absence raises several questions, which should be addressed in future studies. Does the absence of the blue-light-sensitive PR in the compound eye of most ant species reflect an adaptation to the ants' ecological demands or is it actually present but not detected by the limited technical capabilities of the earlier research period?

## 4. Visual pigments

The spectral sensitivity of a PR is mainly determined by its expressed visual pigment. The pigment consists of an opsin protein, which belongs to the G-protein-coupled transmembrane receptor family [45], and a chromophore, which is covalently linked to it. Only the chromophore is able to absorb a light quantum and use its energy to isomerize from the *cis*- to the *trans*-conformation. This leads to a conformational change of the protein and finally to a change of the membrane potential of the PR. Since this phototransduction process is independent of the energy of the absorbed photon (and thus wavelength of light), the spectral sensitivity of a PR is only mediated by the probability of absorbing a photon of a particular energy and wavelength. Photons of high energy (corresponding to UV light) have the highest probability of being absorbed by the chromophore, but the opsin protein can modify this probability (which is called spectral tuning) and thus create PRs with peak sensitivities ranging from UV to green light [46]. However, since the information on the photon energy is lost during this process, the wavelength and intensity of incident light cannot be disentangled, thus an insect with only one PR type cannot discriminate between the light of different wavelengths independent of intensity [12]. As noted earlier, such discrimination requires comparisons between at least two spectrally distinct PR types.

In hymenopteran compound eyes, the rhabdomeric opsins belong to three clades: LW, SW and UV, giving rise to PRs which have peak sensitivities falling into the green (greater than 500 nm), blue (400–500 nm) and UV (less than 400 nm) wavelength range, respectively [7,47]. In ants, only a few molecular studies have been published (figure 1). Of the earliest, Popp *et al*. [48] and Smith *et al*. [49] cloned two opsin cDNAs from *Cataglyphis bombycina* and *Camponotus abdominalis*, belonging to the UV and LW clades of insect opsin genes. Twenty years later, Yilmaz *et al*. [50] identified three opsin genes in the genome of *Camponotus rufipes*, which were homologues to the three major clades found in insects, the UV, SW and LW clade. The authors showed that all three genes were expressed in the eyes, rendering *C. rufipes* a potential trichromatic species. The expression levels of the three opsins were significantly affected by variables such as age and illumination regime, and thus were influenced by both intrinsic and environmental factors [50]. Also unclear is whether the SW opsin mRNA is actually translated into a protein, which would give rise to a blue-light-sensitive PR, since the closely related species *C. blandus* behaves like a dichromatic species and shows no physiological evidence for such a PR ([51], see above; figure 1).

Recent analyses of the genome and transcriptome of several species of ants (*Linepithema humile*, *Acromyrmex echinatior*, *Camponotus floridanus* and *Harpegnathos saltator*) revealed that all possess three opsin genes corresponding to the UV, SW and LW clade of other insects, and that all genes are expressed at the mRNA level (S Albert, A Yilmaz & J Spaethe 2016, unpublished data; figure 1; see also [47,52] for additional genomic and cDNA opsin sequence data). Unfortunately, no histological data on opsin mRNA or protein localization within the ommatidia in ants are available. In honeybees [53] and bumblebees [54], histological studies revealed different ommatidial types, which differ in the composition of PR cells. They showed that the green-light-sensitive PRs are more frequent than the UV- or blue-light-sensitive ones and that only some of the ommatidia comprise all three PR types. Future studies are needed to clarify the spatial opsin expression in ant compound eyes and to resolve the contradiction between the three opsin genes found in all ant genomes and their dichromatic behaviour (see below).

It must not be forgotten that hymenopterans usually possess two LW opsin genes (LW1 and LW2) of which the latter is only expressed in the ocelli [55,56]. Both paralogue genes were recently found in ants (S Albert, A Yilmaz & J Spaethe 2016, unpublished data; see also [47]). The sensitivity of LW2-expressing PRs in hymenopterans seems to be SW shifted compared to LW1, and Mote & Wehner [42] could show that the green-sensitive PRs in the ocelli of *Cataglyphis bicolor* possess a sensitivity maximum at 506 nm.

## 5. Colour processing in the peripheral and central brain regions

**Figure 2.**
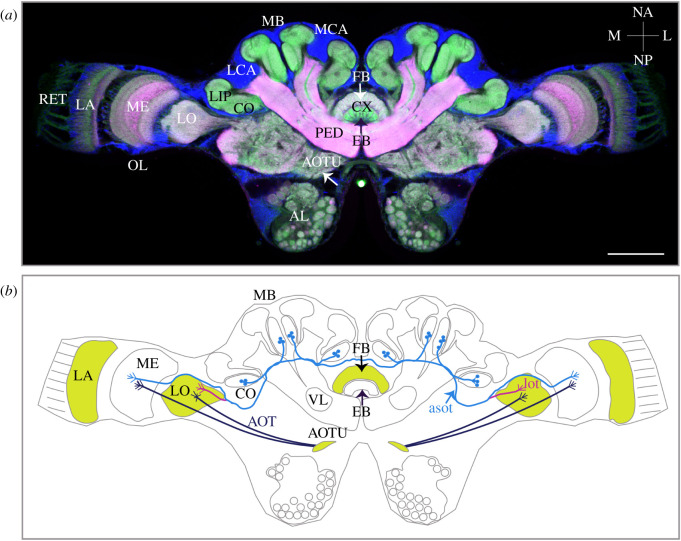
Immunofluorescence labelling and schematic drawing of a *Camponotus blandus* worker brain. (*a*) Frontal views of a central brain with an indication of prominent neuropils and triple-labelled with an antibody to synapsin (red), f-actin phalloidin (green; CF 488 phalloidin, Biotium) and Hoechst nuclear marker (blue; Hoechst 34580, Invitrogen). (*b*) Schematic drawing of a *C. blandus* brain highlighting the visual tracts from the optic lobes. Projections from the optic lobes to the mushroom body calyx are supplied by the anterior superior optic tract (asot, [50]) and lobula tract (lot, [57]) and to the anterior optic tubercle by the AOT. Neuraxes: lateral (L), medial (M), anterior (NA) and posterior (NP). OL, optic lobe; LA, lamina; ME, medulla; LO, lobula; MB, mushroom body; LCA, lateral calyx; MCA, medial calyx; CO, collar; VL, ventral lobe; CX, central complex; FB, fan-shaped body; EB, ellipsoid body; AOTU, anterior optic tubercle; AL, antennal lobe; PED, peduncle; LIP, lip. Scale bar, 100 µm. The neuropils marked in yellow represent the visual processing centres, which showed a volumetric increase after colour learning and memory formation. Please note that the confocal image in (*a*) has been modified by mirroring the optic lobe from the left to the right side since the right optic lobe was damaged during preparation.

Most data on the underlying mechanisms of colour processing comes from studies on *Drosophila*, butterflies and bees, but evidence is compelling that insects in general share many similarities in the morphology and physiology of colour processing [11,12]. For example, visual information received by the PRs in the retina is conveyed to the optic lobe neuropils, the lamina, medulla and lobula (figure 2). The function of the first optic lobe neuropil, the lamina, is mostly related to the response to changing light intensities, enhancement of signal-to-noise ratio and summation [10,58]. Colour-opponent interactions, which are related to colour vision owing to the excitatory and/or inhibitory connections between (at least two) PR classes, are mainly located in the inner layers of the medulla and lobula [11,59]. In some insects, PR terminals [8] and the lamina [60,61] have been shown to contribute to colour processing via spectral opponency (see for a review [11]). The information processed in the optic lobes is relayed to multiple central brain areas, including the anterior optic tubercle (AOTU) [62,63], the anterior and medial protocerebrum, and the mushroom bodies [63–65]. Tracer injections into the medulla and lobula in ants revealed connections between these regions and the mushroom body visual input region, the collar (via anterior superior optic tract or lobula tract) [50,57,66] (figure 2), and two separate areas in the anterior optic tubercle (via anterior optical tract, [50]). The central complex, which is a higher sensory processing and integration centre of the insect brain akin to the mushroom bodies, receives visual sensory information through the anterior optic tubercle. The optic lobe–central complex pathway is particularly conserved and has been shown to integrate celestial cues in the brain of several insects including the ants ([67–69] references therein). As previous studies in ants and in other insects focused mainly on anatomical description of specifically above-mentioned visual pathways, our knowledge of the physiological response of specific neurons to spectral stimuli remains limited, or in the case of ants are completely missing. In one of the studies, the neurons of the anterior optic tubercle in the brain of the locusts have been shown to exhibit colour-opponent responses to unpolarized UV/green light stimuli [69], while the neurons in the central complex did not show any colour-opponent response [70], consistent with findings from central complex recordings in the monarch butterfly [71]. As one of the key processing centres of the celestial compass pathway, the AOTU is suggested to be functionally related to chromatic orientation where spectral variations across the sky are used to derive directional information. The mushroom bodies, on the other hand, have been suggested to participate in more ambiguous tasks such as fine colour discrimination or multi-sensory and contextual learning [10,14,72–74]. In a recent study in butterflies, Kinoshita & Stewart [75] used intracellular recordings to characterize the response of visual input neurons into the mushroom bodies to monochromatic lights. They found three morphologically distinct neurons characterized by a clear colour opponency response [75]. Recently, ants that were trained to discriminate between monochromatic UV and green light showed experience-dependent modifications in the optic lobes, anterior optic tubercle and the upper division of the central complex after colour learning and long-term memory formation [14], suggesting the possible involvement of these neuropils in associative colour vision tasks. Consistent with these findings, several studies performed on other insects suggested a possible role of the central complex and the mushroom bodies in different forms of colour learning and memory formation [13,76,77].

## 6. Behavioural evidence for colour vision

One prerequisite for colour vision is the presence of at least two PR types with different but overlapping spectral sensitivities that are combined by a neuronal mechanism, which compares the input of both types (see above). However, since colour perception is a psychophysical process, it can ultimately only be proven by means of behavioural experiments [2,16].

The honeybee (*Apis mellifera*) is the first invertebrate for which true colour vision could be verified [78] and since then, it has become a model system for investigating the physiology and cognition of trichromatic vision in hymenopterans and other insects [2,79,80]. Behavioural experiments in the context of foraging and nest finding have been conducted to test colour vision in ants but to a lesser extent. Over the past 50 years or so, fewer than a dozen studies have demonstrated that (true) colour vision exists in ants (figure 1; see also [81]). In one of the earliest experiments, Kiepenhauer [82] showed that *Formica polyctena* workers used monochromatic light to orient themselves to their nest entrance. In a more recent study, Carbaugh *et al*. [83] found that the fire ant *Solenopsis invicta,* in a dual choice experiment, preferred red glass beads over yellow and green over blue when digging a nest. However, these studies did not exclude the possibility that ants might have used achromatic cues for their choices because the control experiments (e.g. for brightness or receptor-specific sensitivity differences) were not performed. Nonetheless, other studies, which used monochromatic light [84–86], LED light [51] or coloured paper [87], could show that the use of colour information was independent of brightness by changing light intensities during training (in most of the studies) or by presenting the colour stimulus together with distractors of different shades of grey [87] (see figure 1). Aksoy & Camlitepe [85], for example, trained *Formica cunicularia* workers to enter a Y-maze, where food was offered in one arm together with a monochromatic light stimulus. In the other arm of the Y-maze, a different light was presented but without any reward. After several training bouts and regular interchanging of the colour stimuli between the two arms, ants were able to discriminate between a UV (370 nm) and a green light (540 nm) even when the intensity of one of the lights was reduced by one log unit, indicating true colour vision. Yilmaz *et al*. [51] conducted similar experiments in a Y-maze with *Camponotus blandus*. They could show that *C. blandus* workers can successfully discriminate between UV (365 nm) and blue light (450 nm) and between UV and green light (528 nm) even when intensity varied by two log units, but the ants failed to discriminate between blue and green light, suggesting dichromacy [51].

In the above-mentioned studies, experiments were performed on freely walking ants that were searching for food or the nest entrance. An established procedure to investigate aspects of colour learning and discrimination in honeybees and bumblebees uses the proboscis extension response of restrained animals [88–91]. It allows for the control of environmental factors and for the use of electrophysiological recordings during the learning process. Recently, a similar protocol was established for restrained ants using the so-called maxilla labium extension response (MaLER) to test for olfactory [92,93] or visual learning [94,95]. However, we know of only a single publication that examined colour vision in restrained ants by means of the MaLER [96]. The authors of that study showed that *Ectatomma ruidum* workers could discriminate between a green and blue LED light irrespective of intensity, and that they correctly responded to the rewarded stimulus even after 24 h, indicating the presence of an early long-term colour memory [96].

Despite clear evidence for (true) colour vision in ants, almost nothing is known about the dimensionality of the underlying colour vision system, i.e. the number of involved PR types in colour processing. The number of spectrally distinct PR classes in the compound eye is generally used as a proxy for dimensionality, but without behavioural experiments, this cannot be ensured [2]. For example, the Japanese yellow swallowtail butterfly, *Papilio xuthus*, possesses eight different PR types in its compound eyes but uses only a subset of them for colour vision [97]. Ant species with two spectrally distinct PR types are potentially dichromats, and species with three PR types potentially trichromats, but more behavioural data are needed to confirm the link between the number of PR types and the dimensionality of colour vision.

## 7. Concluding remarks

Ants exhibit remarkable capabilities for visual learning and orientation [98–101]. They can learn and memorize simple and complex visual associations through individual experience and adjust their behaviour accordingly [24,51,99,102]. However, most of our knowledge on ant colour vision is derived from early behavioural and physiological studies. To the best of our knowledge, not a single species of ant has been investigated at all crucial levels (opsin genes, PR spectral sensitivities and colour discrimination experiments). Moreover, the published data fails to provide a consistent picture (see figure 1). For example, the colour discrimination capability in *Camponotus blandus* clearly suggests dichromacy [51], whereas in its sister species, *C. floridanus* and *C. rufipes,* all three opsin types were found to be expressed, which could potentially entail the capability for trichromacy (figure 1). Unfortunately, receptor sensitivity data for the genus are completely unexplored. Furthermore, studies have yielded ambiguous results for the same species. For example, physiological recordings in *Cataglyphis bicolor* revealed two PR types, one with peak sensitivity in the green part of the light spectrum and the other in the UV part [41,42], but behavioural studies suggested both dichromacy [103] or even tetrachromacy (although rather unlikely) [86]. Finally, an earlier behavioural study by Marak & Wolken [104] suggested a LW PR with peak sensitivity at 620 nm in *Solenopsis saevissima*, which is very unlikely, since no evidence at the physiological or molecular level for such a red-light-sensitive receptor in ants exists. We therefore advocate that focus should be placed on a few (model) species in which all levels of colour perception could be investigated. Similarly, comparative investigations of the underlying mechanisms of colour vision in diurnal and nocturnal species would be particularly helpful for understanding not only the plasticity of sensory systems in insect brains but also the (ecological) drivers that form them. Since ants, in contrast to most other hymenopterans, do not fly (except for queens and males) and are the only Apocrita where dichromacy has been proven so far, understanding how colour vision evolved in ants will therefore allow a better general understanding of the benefits and costs of colour vision in relation to orientation, foraging and other aspects of a flightless insect life.

## Data Availability

Sequence data on the unpublished ant opsin genes mentioned in figure 1 can be provided upon request.
